# Supplementary material for: A Critical View of the Application of the APEX Software (Aqueous Photochemistry of Environmentally-Occurring Xenobiotics) to Predict Photoreaction Kinetics in Surface Freshwaters
Source: Molecules. 2019 Dec 18;25(1):9. doi: 10.3390/molecules25010009 (PMC7017383; doi:10.3390/molecules25010009)
Supplement: Supplementary file 1 [file molecules-25-00009-s001.zip › 3.2.4_gcc-4.4.0.zip!/3.2.4_gcc-4.4.0/3.2.4_gcc-4.4.0/doc/octave/html/interpreter/Reporting-Bugs.html]

Reporting Bugs - Untitled


Next: Bug Criteria,
Previous: Actual Bugs,
Up: Trouble


---

### E.2 Reporting Bugs

Your bug reports play an essential role in making Octave reliable.

When you encounter a problem, the first thing to do is to see if it is
already known. See Trouble. If it isn't known, then you should
report the problem.

Reporting a bug may help you by bringing a solution to your problem, or
it may not. In any case, the principal function of a bug report is
to help the entire community by making the next version of Octave work
better. Bug reports are your contribution to the maintenance of Octave.

In order for a bug report to serve its purpose, you must include the
information that makes it possible to fix the bug.

If you have Octave working at all, the easiest way to prepare a complete
bug report is to use the Octave function `bug_report`. When you
execute this function, Octave will prompt you for a subject and then
invoke the editor on a file that already contains all the configuration
information. When you exit the editor, Octave will mail the bug report
for you.

— Function File: **bug\_report** ()  
> Have Octave create a bug report template file, invoke your favorite
> editor, and submit the report to the bug-octave mailing list when
> you are finished editing.

- Bug Criteria- Where: Where to send your bug report.- Reporting: How to report a bug effectively.- Patches: How to send a patch for Octave.
